# Rezum therapy, a feasible and safe treatment for the larger prostate

**DOI:** 10.1016/j.ijscr.2021.105871

**Published:** 2021-04-07

**Authors:** Rashed Rowaiee, Aya Akhras, Farhad Kheradmand Janahi

**Affiliations:** a College of Medicine, Mohammed Bin Rashid University for Medicine and Health Sciences, Dubai, United Arab Emirates; b Department of Urological Surgery, Mediclinic City Hospital, Dubai Healthcare City, Dubai, United Arab Emirates

**Keywords:** Rezum, Benign prostatic hyperplasia, Lower urinary tract symptoms, Minimally invasive treatment, Case report

## Abstract

Benign prostatic hyperplasia (BPH) is the leading cause of Lower Urinary Tract Symptoms (LUTS) in men worldwide. Urinary frequency, nocturia and incomplete emptying are common symptoms experienced by men with BPH, which significantly reduce their quality of life. Treatment for this dysfunction includes surgical and non-surgical modalities such as watchful waiting, lifestyle modification and medication. Historically, Transurethral Resection of the Prostate (TURP) had been considered the gold standard mainstay of treatment; however, in recent years, minimally invasive techniques which offer similar outcomes, fewer complications and cost-effectiveness have gained popularity. Rezum, is a relatively new minimally invasive modality used to treat BPH which has been recommended for prostate volumes of ≤80 cc. Herein, we present a successful case of managing BPH in a middle-aged patient with a prostate volume of 186 cc.

## Highlights

• Rezum is a novel approach to treating BPH symptoms in patients with prostates <80 cc while maintaining sexual function.
• Based on our case, Rezum may also be considered as a MIT for BPH in patients with larger prostates, with a good safety profile.

## 1 Introduction and importance

Middle aged men routinely present to the urology clinic with lower urinary tract symptoms, usually in the context of undiagnosed benign prostatic hyperplasia. Symptoms include hesitancy, intermittent stream, prolonged micturition, frequency, feeling of incomplete bladder emptying and nocturia, all of which can be quite bothersome to these patients and significantly impacts their quality of life [1]. Treatment for BPH ranges from lifestyle modifications to medical and surgical treatments [2]. Medical treatments usually involve alpha blockers, 5-alpha reductase inhibitors and phosphodiesterase 5 inhibitors; all of which come with a myriad of side effects including, but not limited to, orthostatic hypotension and sexual dysfunction. Surgery has always been a last resort for patients with BPH, with TURP being the gold standard approach. However, this procedure does come with a hefty price in which intra-op bleeding and sexual dysfunction being common side effects. Recently, there has been some progress in introducing novel minimally invasive treatments, which have similar outcomes while maintaining a low risk profile and avoiding the side-effects incurred by TURP [3].

Rezum, a modality that makes use of steam injections via transurethral access into the transitional prostatic zone to thermally ablate tissue using a minimally invasive approach has been increasing in popularity [4]. Its main strengths lie in its safety profile, minimal sexual side effects, minimal anesthetic needs and cost [5]. Although it has proven its effectiveness and popularity among patients, it is currently only recommended and used on small prostates (< 80cc) [5]. Rezum, in theory, can be a viable option for men with large prostates, though this is a largely unexplored area of interest. We report the use of Rezum to treat BPH symptoms in a middle-aged man with a prostatic volume of > 180 cc. This case report has been reported in line with the SCARE Criteria [6].

## 2 Case presentation

A 56-year-old father of 8 with well-controlled hypertension presented to the urology clinic in January of 2020 with a chief complaint of worsening nocturia, urinary frequency, weak stream and post micturition dribbling. He is a known case of BPH which first presented in 2015 and had undergone all the necessary investigations to arrive at the diagnosis including an initial prostatic biopsy to confirm the diagnosis of BPH. On further questioning, there was no significant family history noted or any inheritable diseases. Currently, our patient is working at an office job and leading a mostly sedentary lifestyle, he is a lifelong non-smoker and denies use of alcoholic beverages or recreational drugs. At the time, his prostate volume measured 65cc with a peak urinary flow rate (Qmax) of 4.8 mL/s, post void residual (PVR) of 30 mL and a total PSA of 2.1 ng/mL. He was advised to perform a few lifestyle modifications in relation to fluid and caffeine intake, in addition to starting medications to improve his symptoms. Subsequently, started taking Alfuzosin, Dutasteride and Solifenacin (Table 1).

**Table 1:** Patient lab results at first visit, pre-Rezum and post-Rezum.

	2015	January 2020	September 2020 (3-months post-op)
Total PSA (ng/mL)	2.1	2.6	
Prostate volume (cc)	65	186	170
Qmax (mL/s)	4.8		17.5
PVR (mL)	30	121	45

He had inconsistently maintained this regimen for a period of 4 years while following up with the urologist frequently. In 2018, he had stopped his medication due to unwanted side effects after. In January of 2020, the patient presented to the urology clinic with complaints of worsening symptoms, especially worsening nocturia. At this time, his PSA was 2.6 ng/mL, PVR had increased to 121 mL on ultrasound and an estimated prostatic volume of 186cc, with an impression of large median lobe projecting into the bladder space. Discussion with the patient revolved around the need of a surgical intervention to alleviate his symptoms, as medical management was less likely to offer further benefit. The patient was hesitant to undergo TURP due to the risk of sexual dysfunction and retrograde ejaculation. Hence, Rezum was deemed more reasonable for the patient due to its low risk profile.

**Fig. 1:**
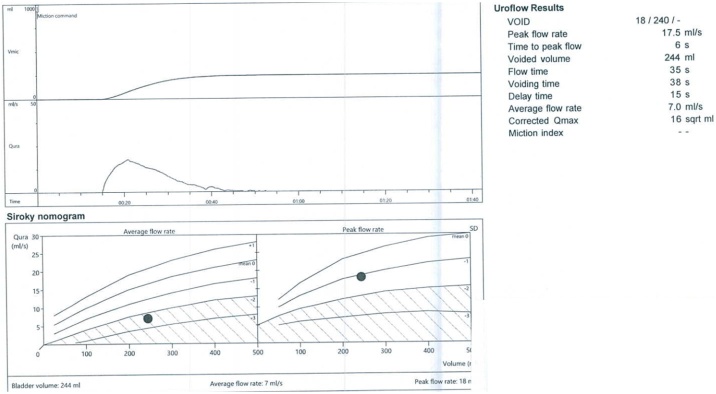
Post-Rezum Uroflowmetry showing Qmax.

**Fig. 2:**
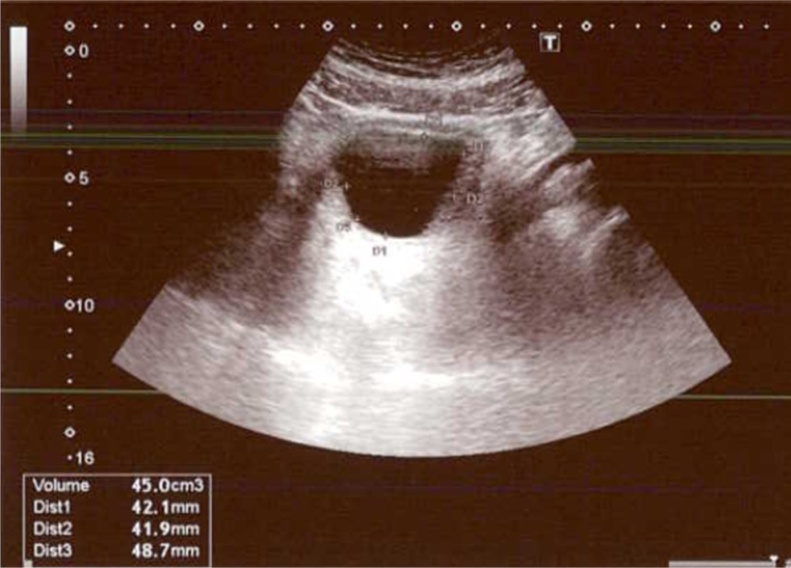
Post-Rezum bladder Ultrasound showing PVR.

In July of 2020, the procedure was done in the operating theatre under general anesthesia by a skilled urologist, which was under the request of the patient, in which 4 treatments were administered to the left lobe of the prostate with 3 further treatments in the right lateral lobe. A two-way 22f foley catheter was then inserted. Total operating time was 15 min with 0 mL estimated blood loss. Foley’s catheter was then removed at 6 days post-op. At 3 months follow up, all medications were stopped and uroflowmetry results showed a Qmax of 17.5 mL/s and a PVR of 45 mL (Fig. 1, Fig. 2). Ultrasound of the prostate estimated prostatic volume at around 170cc. Upon further follow up, the patient added that the frequency of nocturia had reduced to only once per night, with improvement in his sexual function as compared to his pre-op state.

## 3 Clinical discussion

Our case report highlights the successful treatment of BPH via the Rezum procedure in a patient with a prostate size of 186cc, larger than the recommended treatment size (30–80 cc). Rezum is considered to be a breakthrough in the treatment of BPH, by providing a quick, safe and cost-effective option for patients [7]. Conventional medical therapy with alpha blockers or 5-alpha reductase inhibitors are usually recommended for first line therapy, however a high 1-year non-compliance rate has been noted due to inadequate resolution of symptoms or side effects [8]. Moreover, TURP has been offered as a surgical treatment for BPH and LUTS for many years, however its effect on sexual function, need for regional and general anesthesia, as well as consideration in patients with multiple comorbidities make newer techniques amenable to consideration. Rezum has many advantages over medical treatment or TURP, including lower rates of sexual dysfunction or bleeding.

Rezum water vapor thermal therapy is approved for treating LUTS in patients with BPH, particularly in prostates less than 80cc. It has the advantage of preserving sexual function, compared to TURP. However, due to its novelty and the paucity of data around its efficacy among different patient populations, the data remains scarce regarding its use in treating prostates over 80 cc’s.

The American Urological Association Guidelines have added 2 minimally invasive treatments for surgical management of LUTS due to BPH: Rezum therapy and Prostatic Urethral Lift [9]. These options were recommended for men wishing to preserve sexual function. However, Miller et al’s study drew indirect comparisons of these 2 procedures, detailing increased symptom improvement, lower retreatment rates and lower cost with Rezum therapy [10].

From our case, we postulate that Rezum may be an effective treatment modality for treating LUTS due to BPH in a larger prostate. We observed marked improvement in subjective and objective treatment measures such as symptom reporting, post void residual and peak urinary flow rate. Moreover, the reduction in PVR and Qmax we observed is similar to that noted in other studies with smaller prostate volumes [11]. Johnston et al. reported 4-year outcomes in patients treated with Rezum in the United Kingdom therapy, detailing high satisfaction rates, significantly improved Qmax, quality of life score (QoL), IPSS scores, and erectile function questionnaire scores. McVary et al. observed similar outcomes at 1 year follow up [7]. Our study has the limitation of no measured IPSS or QoL pre and post operatively, which would further help quantify improvement. However, their study population contained patients with prostates 30–80 cc, while our case report presents similar satisfactory outcomes in a patient with a larger prostate volume of 186cc. It is noteworthy to assess the degree of improvement observed in our patient in terms of PVR and Qmax post operatively. We have noted a decrease of 76 mL in PVR 3-months post operatively while the Qmax has elevated considerably compared to initial presentation. This is in line with other published data focusing on larger prostates [12].

In a systematic review and meta-analysis by Miller et al. of 514 patients treated with Rezum therapy, IPSS, IPSS_QoL and Qmax significantly improved at all follow up intervals, up to 4 years [10]. The most common adverse events were dysuria, urinary retention and urinary tract infection in a small percent of patients. These side effects are less morbid and transient, compared to those observed with TURP, such as; retrograde ejaculation, urinary tract infections and incontinence [9], Our patient did not report or experience any urinary tract infections, hematuria or sexual dysfunction which were noted in other studies [11]. In addition to low complication rates, Miller et al. reported low retreatment rates at 4 year follow up (7%).

While the data regarding the use of Rezum in larger prostates (>80cc) is scarce, the available literature reports on successful achievement of symptom resolution and increased quality of life. Garden et al. reported on the use of Rezum therapy in patients with prostates >80cc [12]. Their data revealed improved Qmax and PVR post operatively as well as improved symptom scores. Moreover, Bole et al. reported on Rezum post-operative outcomes in patients with large versus small prostates (<80cc) [13]. Their data revealed similar objective and subjective symptom improvement in peak flow rates, post-operative catheter free rate and a similar complication rate among both patient populations undergoing surgery. From this data, we encourage further large-scale comparative long-term studies regarding the effectiveness of Rezum therapy in men with larger prostates, focusing on satisfactory outcomes compared to conventional surgery or medical treatment.

From this case report, we can begin to understand the virtue and convenience that Rezum offers, especially among patients with prostates larger than the recommended threshold of 80cc. Innovative and novel management options are key stepping stones to the advancements in medicine and surgery. Understanding their efficacy in varied patient population can assist physicians in understanding and personalizing care, in order to provide the best care and treatment options or their patients.

## 4 Conclusion

In this case report, we aimed to highlight the benefit, efficacy and safety of offering Rezum to symptomatic patients with prostate volumes of > 80cc. Rezum has proven to be an efficient, and minimally invasive method for the treatment of BPH. It is associated with similar outcomes and improvement in quality of life, while carrying a low risk profile. Although Rezum is currently being offered to men with small prostates, our experience suggests that it may also be a viable modality to those with large prostates. We hope that this experience encourages further research into the use of Rezum for prostates larger than 80cc.

## Declaration of Competing Interest

All authors declare that there is no conflict of interest.

## Funding

No author had received any form of funding for this project.

## Ethical approval

This project is exempt from ethical approval.

## Consent

Written informed consent was obtained from the patient for publication of this case report and accompanying images. A copy of the written consent is available for review by the Editor-in-Chief of this journal on request.

## Author’s contribution

Rashed Rowaiee – Data collection, writing the paper.

Aya Akhras – Writing the paper.

Farhad Janahi – project overview.

## Registration of research studies

Not Applicable.

## Guarantor

Rashed Rowaiee, Aya Akhras and Farhad Janahi.

## Provenance and peer review

Not commissioned, externally peer-reviewed.
